# Bee breweries: The unusually fermentative, lactobacilli-dominated brood cell microbiomes of cellophane bees

**DOI:** 10.3389/fmicb.2023.1114849

**Published:** 2023-04-05

**Authors:** Tobin J. Hammer, Jordan Kueneman, Magda Argueta-Guzmán, Quinn S. McFrederick, Lady Grant, William Wcislo, Stephen Buchmann, Bryan N. Danforth

**Affiliations:** ^1^Department of Ecology and Evolutionary Biology, University of California, Irvine, Irvine, CA, United States; ^2^Department of Entomology, Cornell University, Ithaca, NY, United States; ^3^Smithsonian Tropical Research Institute, Panama City, Panama; ^4^Department of Entomology, University of California, Riverside, Riverside, CA, United States; ^5^Department of Soil and Crop Sciences, Colorado State University, Fort Collins, CO, United States; ^6^Department of Entomology, The University of Arizona, Tucson, AZ, United States; ^7^Department of Ecology and Evolutionary Biology, The University of Arizona, Tucson, AZ, United States

**Keywords:** bacteria, symbiosis, microbiota, *Apilactobacillus*, Colletidae, Diphaglossinae, *Ptiloglossa*

## Abstract

Pathogens and parasites of solitary bees have been studied for decades, but the microbiome as a whole is poorly understood for most taxa. Comparative analyses of microbiome features such as composition, abundance, and specificity, can shed light on bee ecology and the evolution of host–microbe interactions. Here we study microbiomes of ground-nesting cellophane bees (Colletidae: Diphaglossinae). From a microbial point of view, the diphaglossine genus *Ptiloglossa* is particularly remarkable: their larval provisions are liquid and smell consistently of fermentation. We sampled larval provisions and various life stages from wild nests of *Ptiloglossa arizonensis* and two species of closely related genera: *Caupolicana yarrowi* and *Crawfordapis luctuosa*. We also sampled nectar collected by *P. arizonensis*. Using 16S rRNA gene sequencing, we find that larval provisions of all three bee species are near-monocultures of lactobacilli. Nectar communities are more diverse, suggesting ecological filtering. Shotgun metagenomic and phylogenetic data indicate that *Ptiloglossa* culture multiple species and strains of *Apilactobacillus*, which circulate among bees and flowers. Larval lactobacilli disappear before pupation, and hence are likely not vertically transmitted, but rather reacquired from flowers as adults. Thus, brood cell microbiomes are qualitatively similar between diphaglossine bees and other solitary bees: lactobacilli-dominated, environmentally acquired, and non-species-specific. However, shotgun metagenomes provide evidence of a shift in bacterial abundance. As compared with several other bee species, *Ptiloglossa* have much higher ratios of bacterial to plant biomass in larval provisions, matching the unusually fermentative smell of their brood cells. Overall, *Ptiloglossa* illustrate a path by which hosts can evolve quantitatively novel symbioses: not by acquiring or domesticating novel symbionts, but by altering the microenvironment to favor growth of already widespread and generalist microbes.

## Introduction

Bees are a diverse group of key pollinators (Michener, 2007; Danforth et al., 2019). As many species are declining (Ollerton et al., 2014; Goulson et al., 2015; LeBuhn and Vargas Luna, 2021), there is an urgent need to understand ecological factors that influence bee health. One such factor is the microbiome, the assemblage of beneficial, neutral, and harmful microbes associated with a host. Though molecular studies of bee microbiomes began over 15 years ago (Jeyaprakash et al., 2003; Mohr and Tebbe, 2006), and culture-based studies much earlier (Batra et al., 1973; Gilliam, 1979), our understanding of bee microbiomes is far from comprehensive. Only a small fraction of the ~20,000 described bee species (Michener, 2007) have been microbially characterized to date. The majority of studies are on the social corbiculate bees and a handful of solitary bee groups (Voulgari-Kokota et al., 2019). Moreover, as bees are holometabolous and nest-dwelling insects, multiple life stages and microhabitats need to be characterized for a complete picture of bee-microbe interactions. The brood cell, which contains pollen provisions and larvae, is much harder to sample than adults, but has a central role in bee biology. In social corbiculate bees, larvae and adults have very different microbial associations (Kwong and Moran, 2016). In solitary bees—which make up the majority of bee diversity (Danforth et al., 2019) — brood cell microbiome composition and function are poorly understood.

16S rRNA profiling-based studies have provided an initial picture of microbiome composition in solitary bee brood cells. Lactobacilli tend to be dominant members of the bacterial community, across distantly related bee species (McFrederick et al., 2012, 2017; Kapheim et al., 2021). However, for large swaths of bee diversity—including entire families—brood cell microbiomes remain uncharacterized. Moreover, potential variation in the absolute abundance and activity of microbes cannot be inferred from 16S rRNA profiles (Hammer et al., 2019). Here, the decades of research on bee nesting biology provide some clues. In many bee taxa, larval provisions are semi-solid or dough-like, and sometimes quite dry (Danforth et al., 2019; Cane and Love, 2021). Microbial growth in these substrates could be limited by low water potential (Bartlett and Roberts, 2000), as well as other factors. However, in the family Colletidae, provisions are liquid or semi-liquid (Rozen, 1984; Michener, 2007; Almeida, 2008; Sarzetti et al., 2013). The brood cell of colletid bees is lined by an impermeable “cellophane” coating, produced by the Dufour’s gland, that both protects the larva during development and prevents the liquid provisions from leaking into the surrounding substrate (Rozen, 1984; Almeida, 2008; Danforth et al., 2019). Unusually for solitary bees, the colletid subfamily Diphaglossinae also have open brood cells, which have been suggested to facilitate gas exchange (Roberts, 1971). Brood cell venting could be particularly important when there are large numbers of metabolically active microbes alongside the developing bee larva.

Within Diphaglossinae, the genus *Ptiloglossa* has provisions that are particularly watery, and have an obvious odor and taste characteristic of fermentation. This trait was first observed by D. H. Janzen in Veracruz, Mexico in the early 1960s (pers. comm.) and reported by Roberts for a Costa Rican species, *P. costaricana*, in 1971 (Roberts, 1971). Vigorous microbial growth in healthy brood cells appears to be characteristic for this genus, and not a case of sporadic microbial spoilage (which is not uncommon among bees; Batra et al., 1973). Strong fermentation odors have also been noted in *Ptiloglossa* brood cells in Brazil and Arizona, United States (Rozen, 1984; de Araujo et al., 2020). *Ptiloglossa*, and perhaps other colletids (Michener, 1960), may have similarities with insects such as *Drosophila*, for which microbes make up an important part of the larval diet (Markow and O’Grady, 2008). But colletid bee brood cells have not been studied using molecular methods, limiting our ability to infer the ecological function and evolutionary history of this symbiosis. There are many open questions. Which microbes grow in the larval provisions? Are they unique species domesticated by the bee host? How do they vary between closely related bee hosts and between habitats? Are brood cell microbes vertically transmitted, or acquired from the environment? How do they vary over host development? And what traits might bees be using to “brew” fermenting larval provisions?

Here we explore these questions using microbiome sequencing of field-collected brood cells of three diphaglossine bee species: *Ptiloglossa arizonensis* and *Caupolicana yarrowi* in Arizona, United States, and *Crawfordapis luctuosa* in western Panama. We used 16S rRNA gene sequencing to characterize the composition of bacteria in a range of microhabitats in which they may contribute to bee biology, and among which they may be transmitted: larval provisions, larvae of different developmental stages, pupae, adult guts, and floral nectar. For a subset of *P. arizonensis* larval provisions, we also sequenced shotgun metagenomes. These data were used to measure the relative abundance of nonbacterial microbes such as fungi, infer ratios of bacterial to plant biomass, and assess strain-level diversity. We also constructed a phylogeny, using assembled 16S rRNA gene sequences, to evaluate host- and habitat-specialization of the dominant lactobacilli.

## Materials and methods

### Collections

We collected a small number of bee and nectar samples from southeastern Arizona, United States from August 25–26, 2018. For convenience we refer to this as the “*Caupolicana* dataset” although two bee individuals of other species were also included. We caught three adult *Caupolicana yarrowi* foraging from *Solanum elaeagnifolium*, near Portal, AZ. An adult *Protoxaea gloriosa* (Andrenidae) and *Ptiloglossa arizonensis* were also collected in the same area, both from *S. elaeagnifolium*. We dissected and stored the entire gut, from crop to hindgut. From each of three flowers from the same *Agave palmeri* individual, collected near Portal, AZ, we sampled nectar using sterile swabs. We sampled larval provisions from two *Caupolicana yarrowi* brood cells (from two separate nests at the same site) near Paradise, AZ. More information on this site is given in Rozen et al. (2019). For one sample, 50 μl of liquid was pipetted from the brood cell. For the other brood cell, a sterile swab was used to collect the larval provisions since they were more viscous. The latter brood cell contained a larva of *Triepeolus grandis*, a brood parasitic bee described in Rozen et al. (2019). All samples were transported to the laboratory in a dry-shipping liquid nitrogen dewar, where they were frozen at −20°C.

On August 28 2019, we collected *Ptiloglossa arizonensis* brood cell samples, all from a single aggregation of nests at ~5,200’ elevation, near Portal, AZ. Brood cells were carefully excavated from the soil matrix, and liquid provisions were pipetted into sterile tubes. As reported previously (Rozen, 1984), provisions (especially in early stages) are stratified into a more nectar-rich and liquid top layer, and a more pollen-dense bottom layer. In two brood cells we collected the upper and bottom layers separately for analysis (PA.LP.2 [upper] and PA.LP.3 [lower]; PA.LP.4 [upper] and PA.LP.5 [lower]). In the others, the entire volume of the provisions was collected and mixed. Brood cells varied in how recently they had been provisioned by the adult female. Some (e.g., PA.LP.7) had no egg or larva and were presumably still in the process of being provisioned. Other brood cells contained an egg (e.g., PA.LP.4, PA.LP.5) or developing larvae (e.g., PA.LP.6), which were also collected. Larvae were sampled at different developmental stages, with fresh weights ranging from 9.8–99.2 mg. Mature larvae were sampled from capped brood cells; these larvae had finished consuming the pollen/nectar provisions. One of these larvae was later (during homogenization in the laboratory) observed to lack any visible trace of pollen, and had therefore likely completed defecation. We refer to this as a prepupa (following: Michener, 2007; Danforth et al., 2019). Other mature larvae still had pollen in their gut. Both developing and mature larvae were rinsed twice in 70% ethanol before storage, in order to remove soil or provisions on the surface. All provisions and larvae were collected in sterile tubes in a dry-shipping liquid nitrogen dewar for transport to the laboratory.

On August 29, 2019, near dawn, we collected seven adult *P. arizonensis* as they exited nests of the same aggregation. Entire guts were dissected; all bees had empty crops, likely because they were just beginning to forage. From August 28–30, 2019, we also obtained *Agave palmeri* nectar and *Solanum eleagnifolium* flowers, as potential microbial inputs into the brood cell. Flowering stalks from three *Agave* plants in the vicinity of the *Ptiloglossa* nest aggregation were harvested and brought to the field station. From each plant, 500 μl nectar samples were collected from replicate flowers (six in total). Whole *S. elaeagnifolium* flowers (four from each of two plants) were collected in Portal. Nectar and flower samples were also stored in the dewar for transport to the laboratory for long-term storage at −80°C, along with the bee samples.

We collected samples of *Crawfordapis luctuosa* from high-elevation premontane rainforest in Western Panama. A single population, in the vicinity of Mount Totumas near Los Pozos, was sampled in both 2019 (adults) and 2020 (brood cells). Adult bees were caught with an insect net while entering and exiting nests and preserved directly in 100% ethanol. To sample larval provisions and immature stages, we carefully excavated brood cells and collected their contents. Provisions were pipetted into sterile tubes. We used sterilized tweezers to remove developing larvae, mature larvae, pupae, and pharate adults (i.e., those which have completed metamorphosis but not emerged from the cocoon). Adult and brood cell samples were collected and stored at −18-20°C. Adult bees were collected into 50 ml tubes with 15 ml of 100% EtOH (to euthanize immediately), whereas brood cell samples were directly frozen; both freezing and ethanol have been shown to be suitable for insect microbiome characterization, and do not introduce significant bias (Hammer et al., 2015).

### Sample prep and sequencing

For the *Caupolicana* and other bee samples collected in 2018, DNA was extracted using the Qiagen PowerSoil DNA isolation kit. The manufacturer’s instructions were followed, with one modification: samples were heated with Solution C1 for 10 min at 65°C in a dry heat block prior to bead beating. We conducted PCRs in duplicate using GoTaq Colorless Master Mix (Promega) and the 16S rRNA gene primers 515F and 806R (Supplementary methods, Table S1) with Illumina sequencing adapters and unique 12-bp barcodes. Amplification was verified by gel electrophoresis. Amplicons were cleaned and normalized with the SequalPrep Normalization Plate Kit (Thermo Fisher Scientific) following the manufacturer’s instructions, and then pooled. Libraries were sequenced on an Illumina MiSeq using a v2 300 cycle kit (paired-end reads, 2 × 150) at the University of Colorado BioFrontiers Institute Next-Gen Sequencing Core Facility. Negative controls were included during both DNA extraction and PCR amplification.

We also used the Qiagen PowerSoil kit to extract DNA from the *Ptiloglossa* and associated plant samples. For liquid samples (provisions, *Agave* nectar), 100 μl was loaded into bead tubes. *Solanum* flowers were added directly. Larvae and adult guts were weighed, homogenized with a pestle in 100 μl molecular-grade water, and up to 100 μl of homogenate was added to bead tubes. Extractions followed the manufacturer’s protocol, including blanks as negative controls. We then prepared amplicon libraries using 16S rRNA gene primers 799F and 1115R that amplify the V5-V6 region (Supplementary methods, Table S1). We have previously used these primers for paired-end sequencing with inline barcodes (Figueroa et al., 2021), as they minimize amplification of plant chloroplasts and mitochondria (Hanshew et al., 2013; Kembel et al., 2014). PCR conditions are detailed in the Supplementary methods. To normalize the amount of DNA in each library, we used SequalPrep normalization plates (Invitrogen) following the manufacturer’s protocol. We combined 5 μl of each normalized library to create a library pool. To remove primer-dimers and excess master mix components, we cleaned the library pool with AMPure XP beads (Beckman Coulter). We checked the quality and concentration of the pooled libraries using a Bioanalyzer (Agilent). Libraries were sequenced on an Illumina MiSeq using the V3 2 × 300 reagent kit at the Genomics Core of UC Riverside.

For gut microbiome characterization of *Crawfordapis* adults, we used whole abdomens, which contain most of the gut—from the crop to the hindgut. DNA for all samples was extracted using the Qiagen PowerSoil DNA isolation kit, following the manufacturer’s instructions, with one modification: adult abdomens were homogenized for two additional minutes using the beads and lysis solution included in the Qiagen PowerSoil kit. Barcoded 16S rRNA primers (V4-V5 region; 515F/926R) were used for PCR amplification (Supplementary methods, Table S1). Amplicons were cleaned using MoBio UltraClean PCR Clean-Up Kit, following the manufacturer’s instructions. Cleaned, normalized and pooled amplicons were submitted for 2 × 250 bp sequencing on an Illumina HiSeq 2500 at the University of New Hampshire.

To characterize bacterial diversity at finer phylogenetic scales, and to characterize non-bacterial organisms, we conducted shotgun metagenomic sequencing for two *Ptiloglossa* larval provisions samples (PA.LP.1 and PA.LP.9). From the genomic DNA used for amplicon sequencing, we constructed metagenomic libraries using the QIAseq FX DNA Library kit (Qiagen). Library prep methods are described in the Supplementary methods. We also included a commercially available microbial community DNA standard (ZymoBIOMICS D6305) as a positive control, and extraction blanks as negative controls. We checked the quality and concentration of the pooled libraries using a 2100 Bioanalyzer (Agilent). Libraries were sequenced on an Illumina NovaSeq at the UCSF Genomics Core using the S4 2 by 150 bp reagent kit.

### Amplicon data analysis

Raw amplicon data are available from NCBI BioProject PRJNA925568. Demultiplexed sequence libraries from all three datasets were processed separately but using the same methodology. For quality control, chimera removal, and read denoising and binning into amplicon sequence variants (ASVs), we used DADA2 with default parameters (Callahan et al., 2016) except for the number of bases trimmed and truncated (see Supplementary methods). To assign taxonomy to ASVs, we trained the QIIME2 sklearn classifier to the primer set used for each bee species in reference to the SILVA 138 SSURef NR99 full-length sequences and taxonomy databases (Quast et al., 2013; Bokulich et al., 2018): 799–1,115 region for *Ptiloglossa*, 515–806 region for *Caupolicana*, and 515–926 region for *Crawfordapis*.

As different regions of the 16S rRNA gene were targeted in the three datasets, they were analyzed separately (but following the same general approach). In each, ASVs with <100 total sequences across all samples were removed, following (Hammer et al., 2020). ASVs classified as chloroplast, mitochondria, or unidentified Eukaryote were removed. Some bee samples had very high proportions of eukaryotic sequences, potentially indicative of relatively low bacterial biomass (Hammer et al., 2017). *Solanum* flower samples were 98.4–99.9% eukaryotic. The *Ptiloglossa* prepupa sequence library was 97.8% eukaryotic, comprising mostly bee sequences. As these samples were left with a low number of bacterial sequences (below the rarefaction cutoff) they were excluded from further analysis. In the *Crawfordapis* dataset, all of the pupal (*N* = 3) and pharate adult (*N* = 4) samples, four mature larvae, and one adult had low bacterial sequence counts and were subsequently excluded. Contaminants were identified with the tool decontam, applying the prevalence-based method (Davis et al., 2018). In the *Ptiloglossa* dataset, six replicate blanks yielded sequences. Decontam identified six contaminant ASVs, which belong to typical reagent- or human-associated taxa (Salter et al., 2014; Eisenhofer et al., 2019): *Ralstonia, Dietzia*, *Staphylococcus*, *Cutibacterium*, *Micrococcus*, and *Streptococcus*. These were removed from the dataset. In the *Caupolicana* dataset, decontam was not able to identify any contaminants because only one blank yielded any sequences. In the *Crawfordapis* dataset, three blanks yielded sequences. Seven ASVs were identified as contaminants and removed: *Escherichia*, *Streptococcus*, *Klebsiella*, *Cutibacterium*, *Lactococcus*, *Corynebacterium*, and *Staphylococcus*. After filtering and contaminant removal, sequence libraries were rarefied (randomly subsampled) to different depths in each dataset: 34957 reads (*Caupolicana*), 1728 reads (*Ptiloglossa*), and 1809 reads (*Crawfordapis*). Finally, we also evaluated taxonomic classifications (using blastn searches) for the most abundant ASVs classified as *Lactobacillus*. This genus has recently undergone a major revision (Zheng et al., 2020), and changes are not yet fully implemented in the SILVA reference database we used.

To visualize microbial taxonomic composition, we summarized each sample’s read counts at the genus level. Only dominant genera—those with >2% mean relative abundance across samples within the dataset—are labeled and colored in the stacked bar plot; all other genera are shown in white. To analyze patterns of beta diversity, we square-root transformed the ASV data table before calculating Bray-Curtis dissimilarities using the vegan package (Oksanen et al., 2022). We visualized these patterns with non-metric multidimensional scaling (NMDS) plots. The effect of sample type on community composition was first tested with a global permutational multivariate analysis of variance (PERMANOVA). Then, pairwise differences were tested using the pairwiseAdonis package, which applies a correction for multiple comparisons. We used the betadisper function in the vegan package to test for potential variation among sample types in within-group dispersion (i.e., heterogeneity). Differences in alpha diversity (Shannon diversity) among sample types were tested with a one-way ANOVA. After a significant global test we then used Tukey *post hoc* tests for pairwise differences.

### Metagenomic and phylogenetic data analysis

Raw shotgun metagenomic data are available from NCBI BioProject PRJNA925568. We removed adapters and quality-filtered metagenomic reads using cutadapt (Martin, 2011), with a minimum read length of 50, and a minimum phred score of 20. Single-sample assemblies were performed using megahit (Li et al., 2015), with a minimum contig length of 1 kb. The assembly from sample PA.LP.1 contained 1880 contigs with an N50 of 3,541 bp. The assembly from sample PA.LP.9 contained 4,410 contigs with an N50 of 1,405 bp. In preparation for binning, we mapped each sample’s reads to its assembly using bowtie2 (Langmead and Salzberg, 2012, 2) with the – very-sensitive-local setting. Overall alignment rates were low (33.3 and 47.2%), possibly because of a large number of plant sequences that were not assembled (see phyloFlash results below). Assemblies were then binned using MetaBAT 2 (Kang et al., 2019). We used checkM (Parks et al., 2015) to evaluate completeness, contamination, and strain heterogeneity of the bins, and GTDB-tk (Chaumeil et al., 2019) to classify them (Table 1). FastANI (Jain et al., 2018), implemented within GTDB-tk, was used to calculate ANI. Two low-quality bins with ≤20% completeness were discarded (quality defined following: The Genome Standards Consortium et al., 2017).

**Table 1:** Metagenome-assembled genomes (MAGs) from two *Ptiloglossa* brood cell samples.

Sample	% Completeness	% Contamination	% Strain heterogeneity	Reference classification	ANI to reference
PA.LP.1	92.62	2.97	90.91	*Apilactobacillus micheneri*	98.54%
PA.LP.9	76.45	0.31	100.00	*Apilactobacillus timberlakei*	99.08%

Completeness and contamination are metrics calculated by checkM that use lineage-specific, single-copy marker genes to evaluate genome quality. Strain heterogeneity measures the contribution of intraspecific diversity (versus heterospecific diversity) to the reported contamination. MAGs were classified using GTDB-tk.

We also used phyloFlash to identify and classify SSU rRNA sequences from the quality-filtered, paired-end reads (Gruber-Vodicka et al., 2020). phyloFlash was run with the SILVA SSU Ref NR99 reference database for classification (Quast et al., 2013) and other default settings. Taxonomic composition of the metagenomes was then measured using the mapping-based phylotypes identified by phyloFlash. To compare the ratio of bacterial to plant sequences between *Ptiloglossa* and other bees, we included data from 84 bee larval provision metagenomes, sequenced as described above. These data represent eight species of Apidae and one species of Andrenidae, with 5–17 replicate samples each: *Amegilla dawsoni*, *Andrena asteris*, *Anthophora bomboides*, *Centris caesalpiniae*, *Centris cockerelli*, *Centris pallida*, *Diadasia australis*, *Melissodes druriella*, and *Xylocopa micheneri* (unpublished data from MA-G, QM, SB, and BD).

To reconstruct a phylogeny of the dominant lactobacilli, we used the full-length sequences assembled by SPAdes (Bankevich et al., 2012) within phyloFlash. Each sample had a single 16S rRNA sequence with high sequence identity to various *Apilactobacillus* species. We collected sequences of the close matches and outgroup taxa from NCBI Genbank. Sequences were aligned using MUSCLE (Edgar, 2004) and manually trimmed in Jalview (Waterhouse et al., 2009). A maximum-likelihood phylogeny was inferred with IQ-TREE^1^ using the Auto substitution model finder (Kalyaanamoorthy et al., 2017) (which determined TVMe+I as the best-fit model) and standard nonparametric bootstraps (Felsenstein, 1985). The tree was visualized using iTol^2^ and rooted on *Holzapfelia floricola* (Zheng et al., 2020).

## Natural history

**Figure 1:**
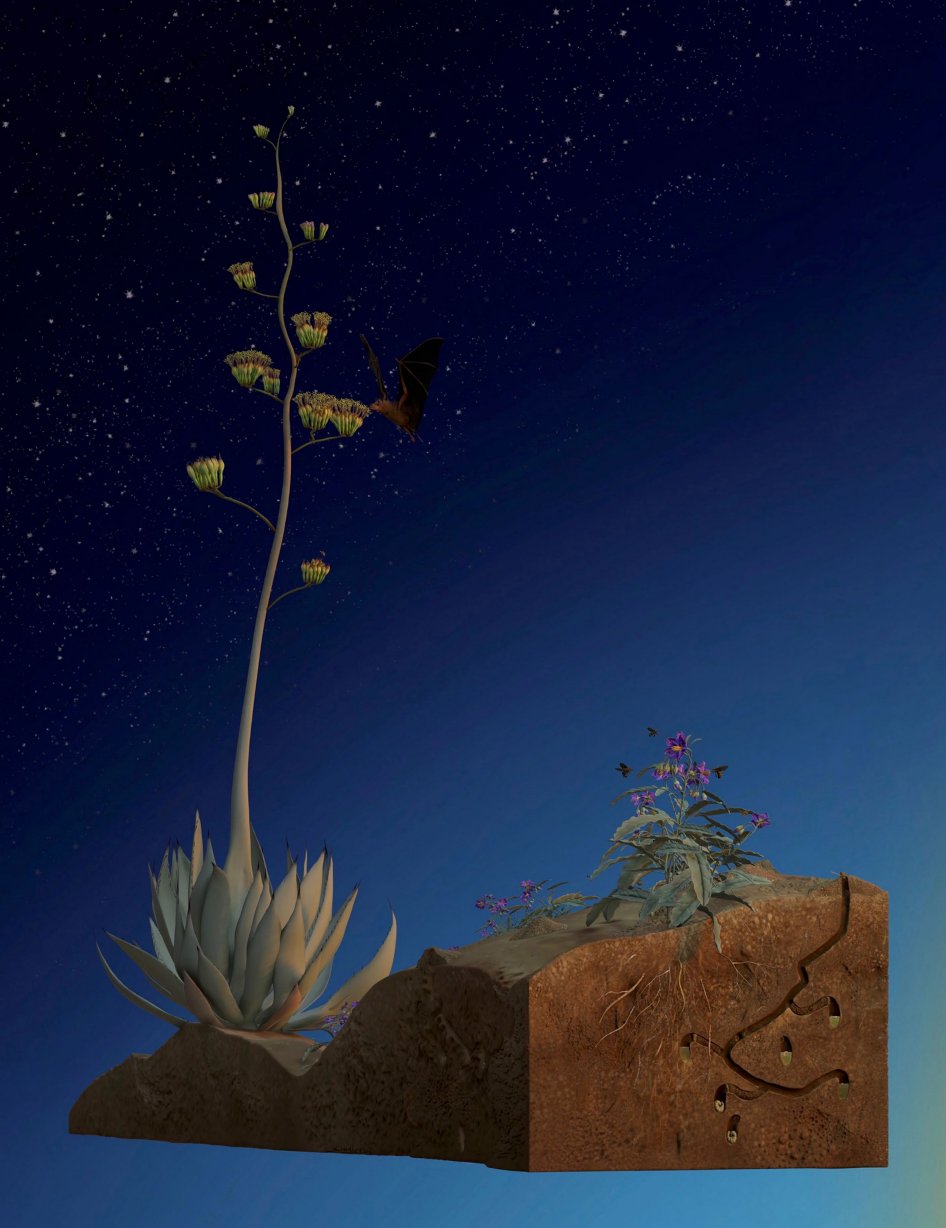
An overview of the ecology of *Ptiloglossa arizonensis* in southeastern Arizona, showing major nectar (*Agave*) and pollen (*Solanum*) sources used to provision the larvae.

Here, we summarize new and published natural history observations relevant to interactions between diphaglossine bees and microbes. At our field site in southeastern Arizona, we observed *Ptiloglossa arizonensis* and *Caupolicana yarrowi* foraging for pollen from *Solanum elaeagnifolium* and *S. rostratum* (Figure 1), matching earlier observations (Linsley, 1962; Rozen et al., 2019). *Solanum* is a commonly reported pollen source across the range of *Ptiloglossa*, though non-Solanaceous plants are also used (Janzen, 1968; Roberts, 1971; Sarzetti et al., 2013). We also have evidence that *P. arizonensis* collects nectar from *Agave palmeri* flowers. First, bees returning from foraging are often dusted with pollen that is morphologically identical to *Agave* pollen. This pollen is concentrated on the dorsum of the mesosoma, a location that matches the position of exserted *Agave* anthers. Second, *Agave palmeri* nectar has a powerful and unique melon (*Cucumis melo*)-like odor. We detected the same odor from *P. arizonensis* brood cells. *Caupolicana yarrowi* were observed collecting nectar from creosote (*Larrea tridentata*). Earlier studies from the southwestern US report that *P. arizonensis* visit *Larrea* as well (Hurd and Linsley, 1975). We do not have information about food plants of the specific *Crawfordapis luctuosa* population we studied, but another population in Western Panama was reported to collect pollen from dozens of flowering plant species, mainly belonging to Solanaceae, Melastomataceae, and Begoniaceae (Roubik and Michener, 1984), all of which (except Begoniaceae species) have poricidal anthers and require buzz pollination (Buchmann, 1983).

The timing and duration of foraging influence the availability and quality of floral resources and the risk of parasitism (Wcislo and Tierney, 2009). These factors could alter the types of microbes to which bees are exposed, and could select for different microbially mediated nutritional or defensive strategies. *Ptiloglossa* are dim-light foraging (crepuscular) bees, with activity concentrated from before, to shortly after dawn (Linsley, 1962; Janzen, 1968; Roberts, 1971; de Araujo et al., 2020). This is the case at our field site in Arizona, where we observed *P. arizonensis* foraging beginning roughly an hour before dawn and ending just after dawn. *Caupolicana* has a longer foraging window. In Arizona, we and others (Rozen et al., 2019) have observed *Ca. yarrowi* foraging later in the morning than *P. arizonensis*, and as late as mid-afternoon; another *Caupolicana* species has been collected near mid-day (Michener, 1966). Our *Crawfordapis luctuosa* study population forages throughout the day, as reported earlier (Roubik and Michener, 1984).

We observed that *P. arizonensis* larval provisions—particularly those in older cells with large larvae—have a sour taste and a strong odor characteristic of microbial fermentation. According to earlier reports from the neotropics, the odor of fermentation is “unmistakable” (Costa Rica; Roberts, 1971), a “strong sour smell” (Brazil; de Araujo et al., 2020) similar to “fermenting beer or mead” (Venezuela; D. H. Janzen, pers. comm.). *Caupolicana yarrowi* brood cells did not have a noticeable fermentation odor, in agreement with published descriptions (Rozen, 1984; Rozen et al., 2019). We observed fermentation odors during collection of *Crawfordapis luctuosa,* although they were not observed in an earlier study (Roubik and Michener, 1984), perhaps due to seasonal or fine-scale temporal differences in fermentation within brood cells. Although these odors are likely to be a useful proxy for overall microbial activity, there are caveats. It is difficult to infer which microbes are abundant, as very different microbes can have overlapping volatile profiles (e.g., *Saccharomyces* yeast and *Lactobacillus* bacteria; Hansen and Hansen, 1994), and as different volatiles have different salience to the human nose. Also, unusual odors in bee brood cells are not necessarily a product of microbial metabolism. They can be derived from plant volatiles or, as in the case of the cheesy-smelling brood cells of *Anthophora*, from glandular substances produced by the bee (Norden et al., 1980).

## Results

**Figure 2:**
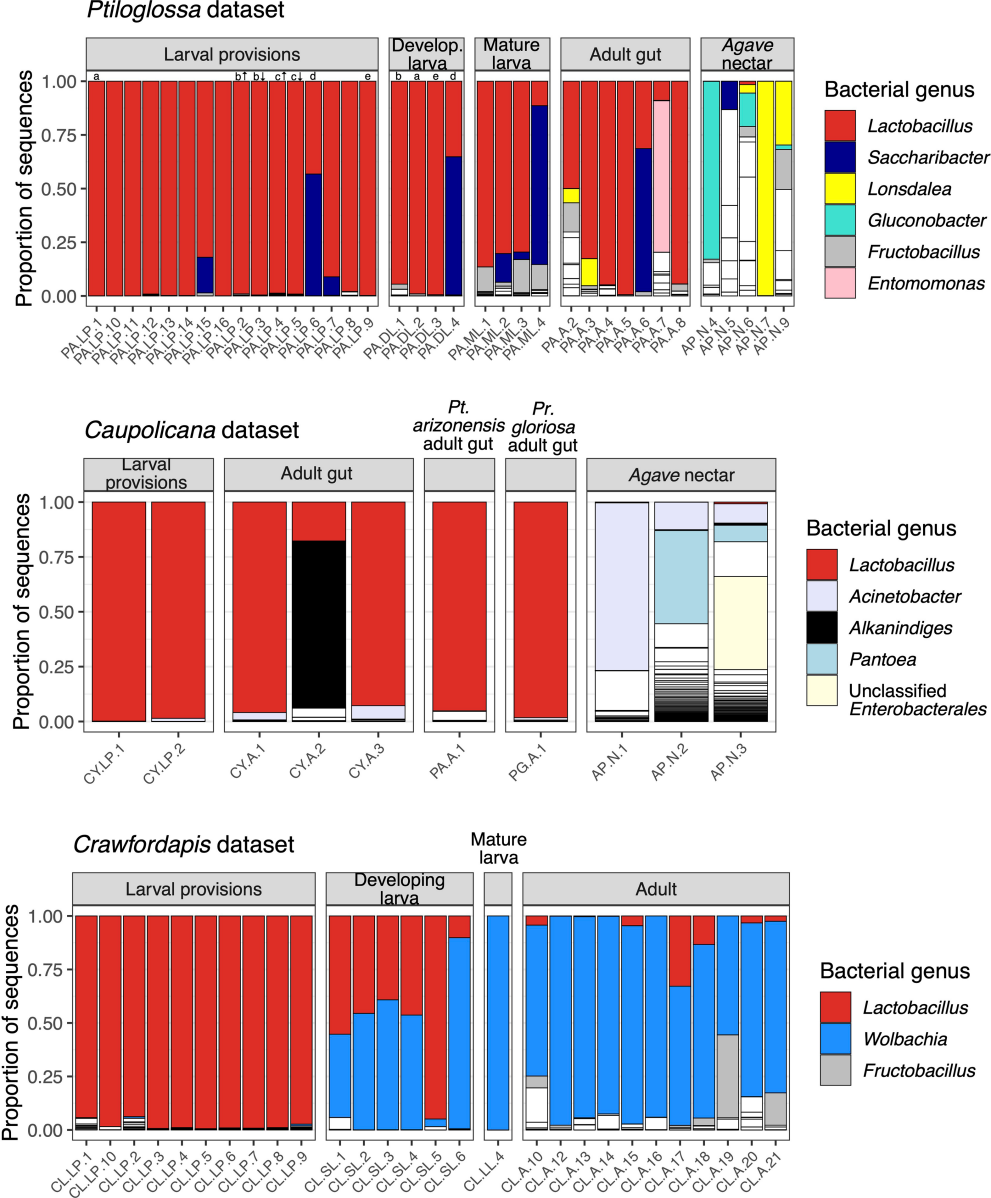
Bacterial composition of bees and nectar. Each column along the x axis is a different sample. Each bar, outlined in black, represents the relative abundance of different bacterial genera within samples. For clarity, only genera with ≥2% mean relative abundance across samples are colored; all others are white. In the *Ptiloglossa* dataset, letters above bars indicate samples that came from the same brood cell (a-e). For two brood cells (b and c), the top (indicated by ↑) and bottom (indicated by ↓) layers were sampled separately. Developing larvae are ordered left-to-right by increasing fresh weight, a proxy for age. In the *Caupolicana* dataset, all samples are of *Caupolicana yarrowi* unless otherwise noted.

Microbiomes of diphaglossine bee larval provisions are dominated by lactobacilli (mean relative abundance +/− SEM: *Ptiloglossa*, 94.3 +/− 3.61%, *N* = 16; *Caupolicana*, 99.2 +/− 0.645%, *N* = 2; *Crawfordapis*, 97.2 +/− 0.627%, *N* = 10; Figure 2). *Lactobacillus* is dominant even in brood cells inferred to have been recently provisioned (i.e., no egg or larva). For two *Ptiloglossa* brood cells in which we separately sampled the top (more nectar-rich) and bottom (more pollen-rich) stratified layers of the larval provisions, we did not observe a difference in microbial composition (Figure 2). In *Ptiloglossa* and *Caupolicana*, the dominant ASVs have 100% sequence identity to various strains of *Apilactobacillus*, particularly *A. micheneri* and *A. timberlakei*. In contrast, *Crawfordapis* larval provisions are dominated by an ASV that may represent a new bacterial species, with <97% identity to isolates belonging to *Lactobacillus*, *Pediococcus*, *Nicolia*, and related genera. For consistency, we use the default classification provided by the SILVA database (*Lactobacillus*) in the text and plots describing 16S rRNA amplicon data. Other bacteria, namely *Saccharibacter*, *Fructobacillus*, and *Acinetobacter*, are only sporadically present, but can be abundant in individual brood cells (Figure 2).

**Figure 3:**
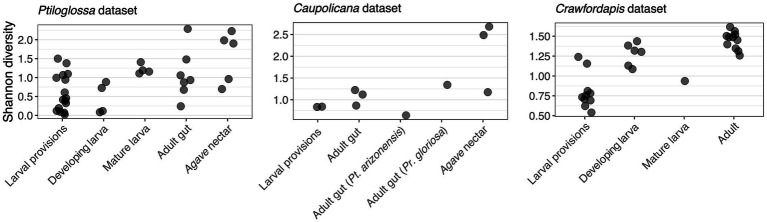
Shannon diversity, a measure of alpha diversity, of bee and nectar-associated bacterial communities. Each point represents a unique sample. In the *Caupolicana* dataset, all samples are of *Caupolicana yarrowi* unless otherwise noted.

**Figure 4:**
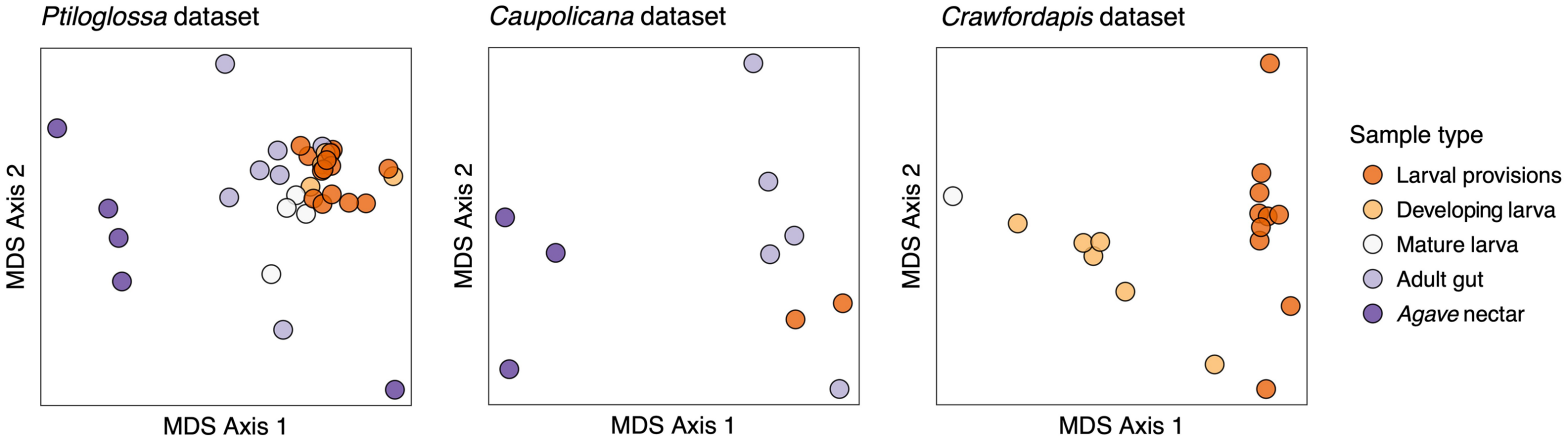
Ordinations (non-metric multidimensional scaling) representing differences in community composition (Bray-Curtis dissimilarities) among samples. Note that in the *Caupolicana* dataset, three bee species are represented within the ‘Adult gut’ group (see Figure 2).

Whole-body microbiomes of developing *Ptiloglossa* larvae are also dominated by *Lactobacillus*, similarly to their diet (compare larvae to provisions samples with matching letters [a, b, d, e] in Figure 2). Secondary bacterial taxa (most notably, *Saccharibacter*) that appear in larval provisions (sample PA.LP.6) also appear at a similar relative abundance in the larva from the same brood cell (sample PA.DL.4; Figure 2), suggesting dietary acquisition. (Larvae of most bees, including diphaglossines, only defecate after they finish feeding and immediately before entering the last larval instar, or prepupal stage (Danforth et al., 2019). These larvae cannot contaminate the provisions through defecation). Microbiomes of *Ptiloglossa* larval provisions and developing larvae are not significantly different in Shannon diversity (*post hoc* test, adjusted *p* = 0.98; Figure 3) or in composition (pairwise PERMANOVA, adjusted *p* > 0.05; Figure 4). In contrast, developing *Crawfordapis* larvae have high relative abundances of *Wolbachia* in addition to *Lactobacillus* (Figure 2), leading to a clear division between larval provisions and developing larval microbiome composition (pairwise PERMANOVA, *R*^2^ = 0.55, adjusted *p* = 0.003; Figure 4) and Shannon diversity (posthoc test, adjusted *p* < 0.01; Figure 3), though not within-group heterogeneity (betadisper, *F* = 0.067, *p* = 0.79).

In *Ptiloglossa*, microbiomes shift, diverging from the diet, as larvae approach the prepupal stage. Mature larvae—those which have consumed all of the provisions, but have not yet defecated—have about twice as high alpha diversity as developing larvae (Figure 3), although this difference is not statistically significant (adjusted *p* > 0.05). (Note that sample sizes are low: N = 4 per sample type.) Mature and developing larvae also appear to have somewhat distinct microbiome composition (Figures 2, 4), though again this difference is not statistically significant (adjusted *p* > 0.05). For *Crawfordapis*, only one out of six mature larvae had enough 16S rRNA reads for analysis. *Wolbachia* is the only bacterium detectable in this larva (Figure 2).

*Lactobacillus*, and potentially all bacteria, are cleared from the gut before larvae enter diapause as a prepupa. We sequenced one *Ptiloglossa* prepupa in which pollen was visibly absent from its gut, confirming that it had already defecated. This sample is nearly devoid of bacteria. 97.8% of the 16S rRNA reads are eukaryotic; based on blastn searches, these are likely to originate from the bee as opposed to plant or other eukaryotic DNA. The remaining bacterial reads do not include any *Lactobacillus*. Rather, these ASVs are all very rare (<100 total reads) or absent in the other *Ptiloglossa* and nectar samples, and hence may be transient or spurious. In *Crawfordapis*, eukaryotic reads are not abundant (<10%) in pupae and pharate (pre-eclosion) adult samples—possibly because of a lower identity to bee rRNA with this primer pair. However, sequencing depth was very low (2–198 reads, mean 47), suggesting low amounts of bacterial DNA in these life stages. *Lactobacillus* is also absent.

Adult *Ptiloglossa arizonensis* collect *Agave* nectar, regurgitating it from the crop into the larval provisions and likely consuming it themselves (see Natural History). Bacterial communities in *Agave* nectar, *Ptiloglossa* adult guts, and *Ptiloglossa* larval provisions are distinct. Shannon diversity is much higher in *Agave* nectar than in larval provisions (adjusted *p* = 0.011; Figure 3). Microbiome composition also differs between each of these habitats (pairwise PERMANOVAs, *R*^2^ = 0.17–0.45, adjusted *p* = 0.01–0.02; Figure 4). As evident in the ordination (Figure 4), within-group microbiome heterogeneity varies among sample types (betadisper, *F* = 2.78, *p* = 0.043), with higher heterogeneity for adult guts and *Agave* nectar. *Lactobacillus* is present in adult guts as well as *Agave* nectar, but at much lower and more variable relative abundances than in larval provisions (Figure 2). In addition to *Lactobacillus*, adult gut microbiomes contain a variety of bacterial genera common among bees and other pollinating insects, such as *Entomomonas* and *Fructobacillus* (McFrederick et al., 2017; Hammer et al., 2020; Wang et al., 2020; Handy et al., 2023). In a smaller set of samples, collected in 2018 from the same site in Arizona, adult guts of *Ptiloglossa*, *Caupolicana*, and *Protoxaea gloriosa* (Andrenidae) were all dominated by *Lactobacillus* (Figure 2). *Alkanindiges*, a gammaproteobacterium detected in other Hymenoptera (Suenami et al., 2019; Koto et al., 2020), was also abundant in one *Caupolicana* adult. *Lactobacillus* and *Fructobacillus* are again dominant bacterial genera in *Crawfordapis* adult microbiomes (whole abdomen samples), alongside *Wolbachia* (Figure 2).

**Figure 5:**
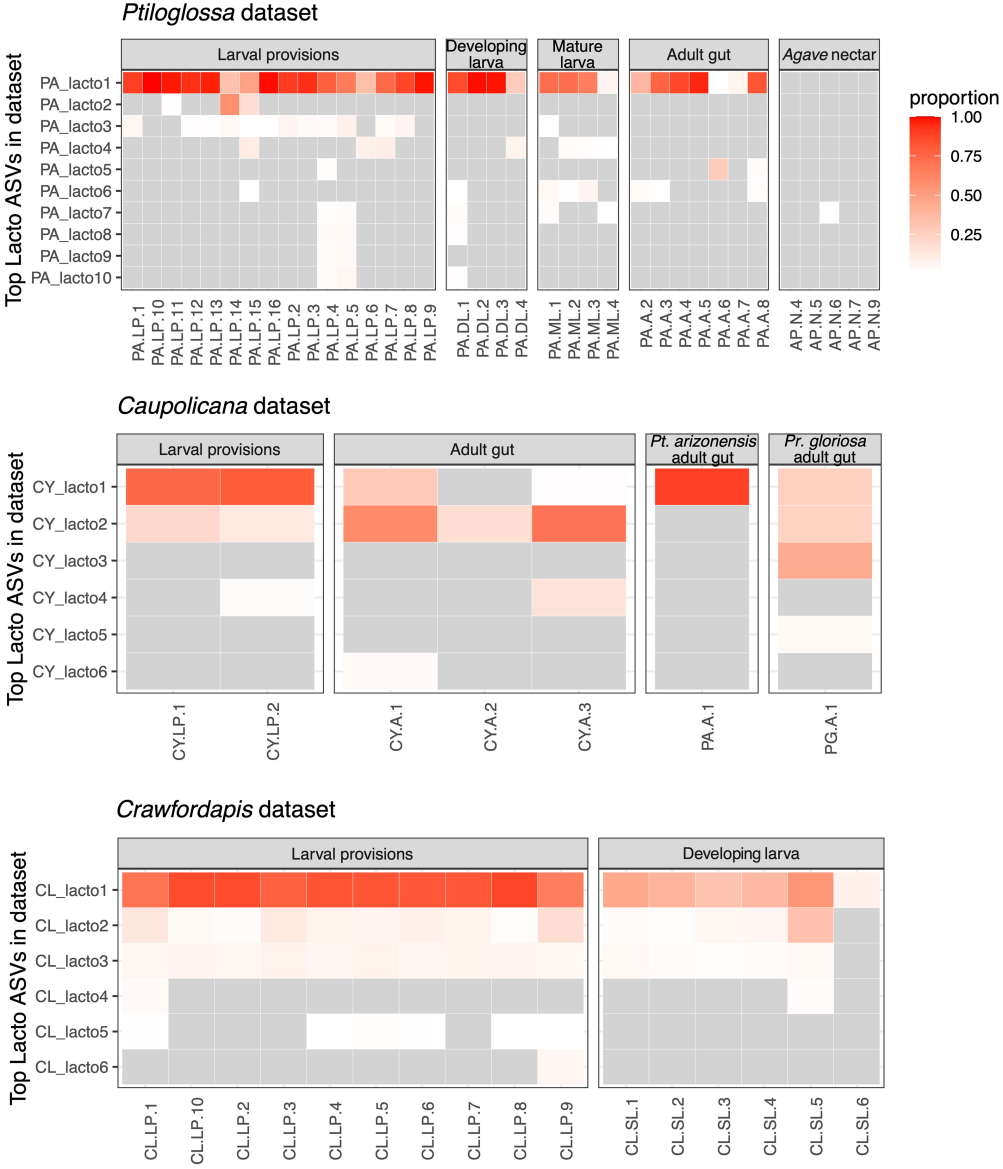
Heatmaps of the dominant ASVs classified as *Lactobacillus* for the three datasets. Columns represent individual bee or nectar samples. Rows represent up to 10 of the top ASVs (ranked by mean proportion across samples). Grey cells represent relative abundance values of <1%. Other cells are colored according to their relative abundance (proportion of sequences). *Agave* nectar is not shown in the *Caupolicana* dataset because no *Lactobacillus* ASVs were detected at ≥1% relative abundance. In the *Caupolicana* dataset, all samples are of *Caupolicana yarrowi* unless otherwise noted.

There are multiple *Lactobacillus* amplicon sequence variants (ASVs) in our three datasets, representing distinct species or strains. We analyzed the distribution of these ASVs to further investigate transmission and potential host-symbiont specificity. In the *Ptiloglossa* dataset, *Lactobacillus* populations across provisions, larvae, and adult guts predominantly belong to a single ASV (Figure 5). However, many of the samples contain one or more additional *Lactobacillus* ASVs. General patterns are similar in the *Caupolicana* and *Crawfordapis* datasets, with 1–2 dominant *Lactobacillus* ASVs (alongside a few rarer ASVs) in provisions, larvae, and adult guts (Figure 5). Within the 2018 *Caupolicana*-focused dataset, we also see evidence of *Lactobacillus* ASV sharing among co-occurring bee species. The dominant ASV in *Ptiloglossa* is present in *Caupolicana*, as well as *Protoxaea gloriosa* (Figure 5), a distantly related bee in the subfamily Oxaeinae, which also produces liquid provisions (Sarzetti et al., 2014).

**Figure 6:**
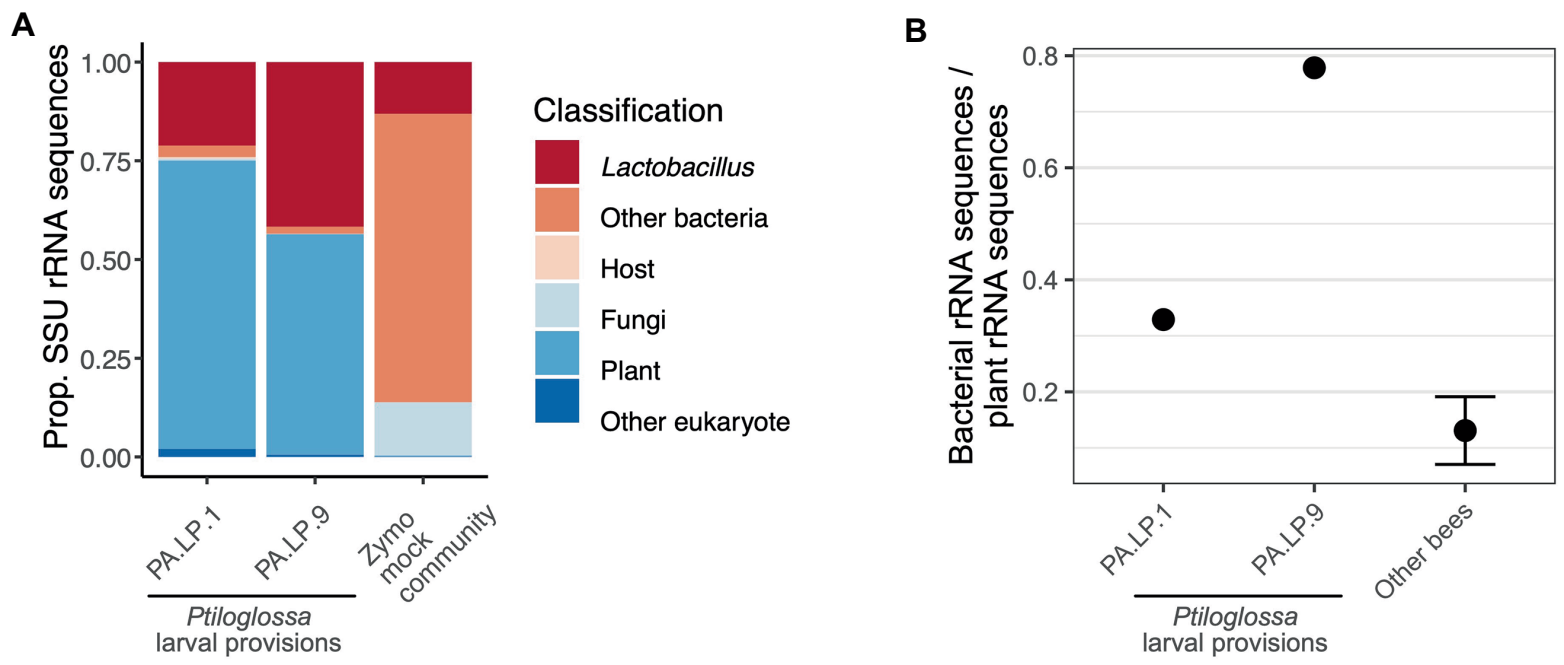
**(A)** Taxonomic composition of small subunit rRNA sequences in shotgun metagenomes of two *Ptiloglossa* larval provisions, and a mock community (Zymo). Sequences classified as “Other eukaryote” lacked any classification below the Domain level and may be artifactual. Note that among *Ptiloglossa* provisions sequences labeled “Other bacteria,” 79% are Lactobacillales (without a lower taxonomic classification). **(B)** The ratio of bacterial:plant rRNA sequences for metagenomes of two *Ptiloglossa* larval provisions, compared with metagenomes of larval provisions of apid and andrenid bees (9 bee species, 84 samples; unpub. data). For the latter, the mean +/− SEM is shown.

Shotgun metagenomic data from larval provisions of two *Ptiloglossa* brood cells corroborate the 16S rRNA gene amplicon data. Based on taxonomic classification of all SSU rRNA genes identified in the metagenomes, microbial community structure is dominated by *Lactobacillus* (Figure 6). Bacterial sequences not classified as *Lactobacillus* mostly (78.7%) belong to the order Lactobacillales; these either belong to related genera or lacked a genus-level classification. We also used these data to quantify the relative abundances of non-bacterial microbes such as fungi. The latter are rare (Figure 6). Fungi occur at 0.502 and 0.120% in the two larval provisions samples. 87.7% of the unidentified eukaryotic sequences—those not classified as host (Metazoa), plant, or fungi—lacked any classification below the Domain level and may be artifactual. *Acanthamoeba* and *Plasmodium* were present, but at very low levels (≤ 32 sequences total). As a control, a mock community (Zymo) was also sequenced and processed alongside the larval provisions. The proportion of *Lactobacillus* in this sample is fairly accurate (13.1% versus expected 12%) and fungi are somewhat overrepresented (13.5% versus expected 4%).

As amplicon sequencing and metagenomics are compositional methods, they do not directly quantify the absolute abundance (biomass) of microbes in a sample. However, variation in the ratio of bacterial to plant SSU rRNA sequences may be used as a proxy for variation in bacterial biomass (relative to plant biomass). We find that the bacteria:plant ratio in *Ptiloglossa* larval provisions is substantially higher than nine non-colletid bee species assayed with the same methodology (Figure 6). This suggests there is likely more bacterial biomass per brood cell in *Ptiloglossa* as compared with many other bee species, in line with inferences of microbial activity based on the presence or absence of fermentation odors. Note it is possible that *Ptiloglossa* females provision brood cells with less pollen than other bees, which would also increase the bacteria:plant ratio.

We further used the shotgun metagenomes to explore sub-ASV-level diversity of lactobacilli in the two *Ptiloglossa* brood cell samples. Single-sample assemblies resulted in one medium-quality and one low-quality metagenome-assembled genome (MAG) per sample (see The Genome Standards Consortium et al., 2017, for quality definitions; Table 1). The medium-quality MAGs are classified as *Apilactobacillus*, matching results from blastn searches of dominant ASVs in the 16S rRNA amplicon dataset. One sample contains an *A. micheneri* MAG, while the other contains an *A. timberlakei* MAG, with high average nucleotide identity (98.5–99%) to previously sequenced genomes (Table 1). As measured using single-copy core genes, bins have high levels of strain heterogeneity (Table 1). This metric evaluates the degree to which reported contamination comes from conspecific strains, versus heterospecifics (Parks et al., 2015). High values suggest that multiple closely related strains were co-assembled into the same MAG. Thus there is likely intrageneric *Apilactobacillus* diversity within each of the two brood cells, including sample PA.LP.9, which contains only a single ASV classified as *Lactobacillus* in the 16S rRNA amplicon data (Figure 5).

**Figure 7:**
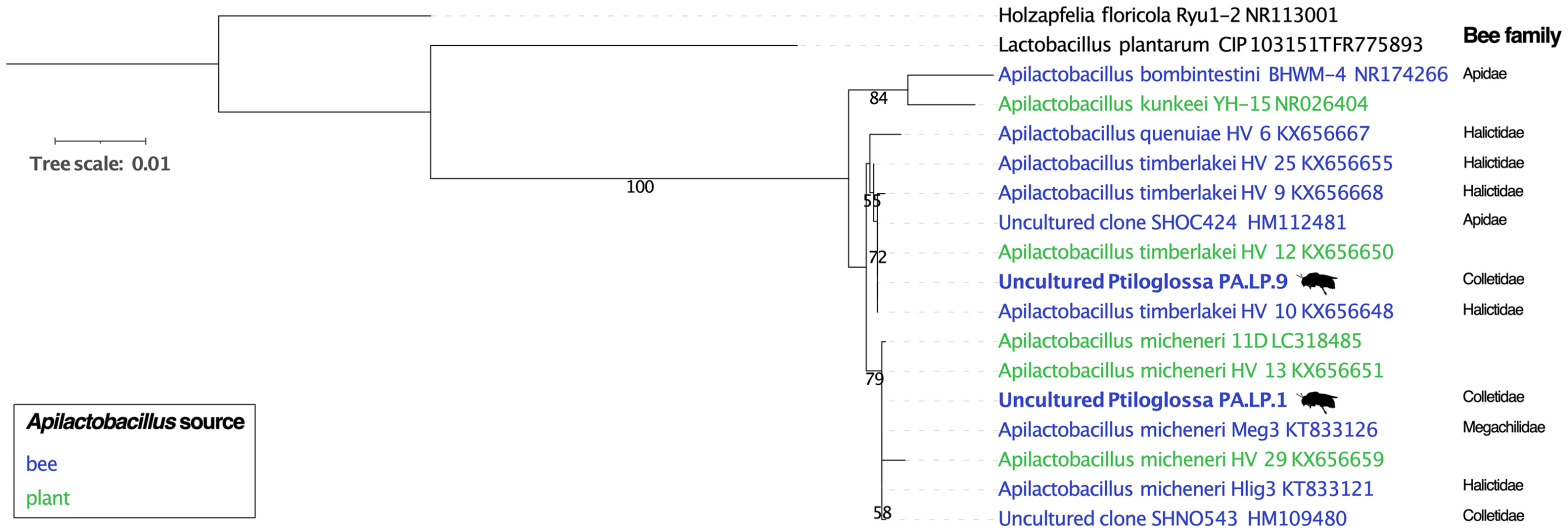
Phylogeny of *Apilactobacillus* and outgroups, including reconstructed 16S rRNA gene sequences from *Ptiloglossa* larval provisions (tip labels in bold, with bee silhouette), and close matches from Genbank. Only >50% bootstrap values are shown. NCBI accession numbers are given in the tip labels. The source (bee versus plant material) from which *Apilactobacillus* were isolated or sequenced is shown. For bee-derived *Apilactobacillus*, the family classification of the source bee is shown.

To investigate possible host-specificity between *Ptiloglossa* and *Apilactobacillus*, we constructed a phylogeny using full-length 16S rRNA gene sequences assembled from the metagenomes. Only a single *Apilactobacillus* sequence was reconstructed from each of the two brood cells using phyloFlash. This does not necessarily conflict with the evidence of within-sample *Apilactobacillus* diversity (Figure 5; Table 1), as the assembler tends to collapse strain-level variability into a single, approximately species-level, consensus sequence (Gruber-Vodicka et al., 2020). Closely matching sequences and outgroups were collected from GenBank. The phylogeny (Figure 7) generally agrees with prior phylogenomic analysis of *Apilactobacillus*, with two sister clades corresponding to *A. micheneri* and *A. timberlakei* (Vuong and McFrederick, 2019). Matching the assembly data (Table 1), one *Ptiloglossa* brood cell (PA.LP.1) has a consensus 16S sequence belonging to *A. micheneri*, while the other (PA.LP.9) has a consensus sequence belonging to *A. timberlakei*. Thus, *Ptiloglossa* exhibit some degree of species-level flexibility in their symbiosis with *Apilactobacillus* (note that these two species are very closely related; Vuong and McFrederick, 2019; Wittouck et al., 2019). Conversely, *Apilactobacillus* are not specialized to particular bee hosts. The *Ptiloglossa*-associated *A. micheneri* has 99.9–100% sequence identity to *A. micheneri* strains isolated from non-colletid bees and from nectar (McFrederick et al., 2018), and to a clone from the gut of a *Caupolicana yarrowi* adult collected in 2006 from southeastern Arizona (Martinson et al., 2011; Figure 7). Likewise, the *Ptiloglossa*-associated *A. timberlakei* has 99.9–100% sequence identity to additional isolates from non-colletid bees and nectar, and to a clone from the gut of a *Diadasia opuntiae* (Apidae) adult bee collected in 2012 from Sonora, Mexico (Martinson et al., 2011; Figure 7). *Diadasia* and *Ptiloglossa* co-occur in Arizona but use temporally non-overlapping floral resources. *Diadasia opuntiae* forage nearly exclusively on cactus (*Opuntia* and *Carnegiea*) flowers for nectar and pollen (Ordway, 1984). Therefore, sharing of lactobacilli between these bees is likely mediated by other flower visitors.

## Discussion

Across all three diphaglossine bee species, in both the Sonoran desert (Arizona, United Sates) and premontane tropical rainforest (Western Panama), microbial communities of larval provisions are near-monocultures of lactobacilli. Larval provisions of *Ptiloglossa* and *Caupolicana* specifically harbor *Apilactobacillus* while those of *Crawfordapis* harbor unique and potentially novel *Lactobacillus*-related taxa. Thus, diphaglossine bees fit an emerging pattern of symbiotic interactions between bees and lactobacilli, spanning solitary species, social species, the adult stage, and brood cells (Kwong and Moran, 2016; McFrederick et al., 2017; Voulgari-Kokota et al., 2019; Tang et al., 2021; Handy et al., 2023).

Consistency of the diphaglossine bee brood cell microbiome is unlikely to come from vertical transmission. Lactobacilli disappear from larvae before metamorphosis—a common phenomenon in gut symbionts of holometabolous insects (Hammer and Moran, 2019). Furthermore, phylogenetic evidence from *Ptiloglossa* brood cells indicates that *Apilactobacillus* are not host-specific; host specificity (or host restriction) is a common feature of vertically transmitted symbionts (Moran et al., 2019). *Apilactobacillus* appear to be frequently exchanged with other bees via flowers, as is the case in other solitary bee-lactobacilli associations (McFrederick et al., 2014, 2017). Brood cell microbiome consistency is likely mediated instead by horizontal transmission coupled with strong ecological filtering (or partner choice; Sachs et al., 2004). *Agave palmeri* nectar (visited by *Ptiloglossa*) does contain detectable levels of lactobacilli, but amid a diverse and heterogeneous background of bacteria. Similarly, a culture-based study in southern Arizona found that *Apilactobacillus* is present in *Agave palmeri* nectar, but at a low abundance relative to other bacteria and yeasts (von Arx et al., 2019). The traits that allow *Apilactobacillus* to colonize brood cell microbiomes are not known, but may include tolerance of low pH, osmotic stress, and oxidative stress, and the ability to metabolize nectar carbohydrates (particularly fructose) and attach to host tissues (Vuong and McFrederick, 2019). Where and when filtering takes place are also unknown, but given that *Apilactobacillus* dominates even freshly provisioned brood cells, one possibility is that it begins in the female bee’s crop. Microbes have been observed at higher densities in the bee crop as compared with nectar (Batra et al., 1973), and the crop of *Xylocopa sonorina* bees is strongly enriched in *Apilactobacillus* (Handy et al., 2023). We cannot directly address this hypothesis because our adult bee samples include the midgut and hindgut in addition to the crop, and these regions may have highly distinct microbiomes (Kwong and Moran, 2016).

Although brood cell microbiomes are homogenous at the level of bacterial genera—*Apilactobacillus* in *Ptiloglossa* and *Caupolicana*, unclassified *Lactobacillus* in *Crawfordapis*—there is diversity at finer phylogenetic scales. Most individual brood cells of all three bee species harbor lactobacilli comprising multiple amplicon sequence variants (ASVs). In *Ptiloglossa*, there is evidence of further diversity not detectable at the ASV level. Across brood cells, *Ptiloglossa* cultures at least two closely related species of *Apilactobacillus*—*micheneri* and *timberlakei*. Within a brood cell, there is also strain-level diversity (Table 1). Thus, ecological filtering in *Ptiloglossa* brood cells is somewhat porous, permissive of multiple species and strains of *Apilactobacillus*. Whether sub-generic diversity of *Apilactobacillus* has functional consequences for bee development is not known. *A. micheneri* and *A. timberlakei* are sister species, and gene sets involved in carbohydrate metabolism generally overlap between their genomes (Vuong and McFrederick, 2019). But there is also evidence for divergence, particularly in genes mediating tolerance of environmental stressors (Vuong and McFrederick, 2019). Partnering with multiple symbiont species and strains can be advantageous to hosts (Batstone et al., 2018). For example, the ability to culture multiple microbial partners with distinct niches could buffer the brood cell symbiosis against environmental variation and against a heterogeneous distribution of *Apilactobacillus* species in flowers. At the same time, bees may not be able to discriminate among closely related lactobacilli. For example, legumes are often unable to selectively exclude certain strains of rhizobia from colonizing root nodules, even those that are ineffective mutualists (Hahn and Studer, 1986; Simms and Taylor, 2002).

Microbiomes of developing *Ptiloglossa* larvae are statistically indistinguishable from those of their diet. Further, microbial variation among provisions is reflected in the corresponding larvae. A similar pattern occurs in caterpillars, where ingested bacterial communities pass transiently through the gut (Whitaker et al., 2016; Hammer et al., 2017). This result suggests that: (i) there are no substantial populations of unique symbionts inhabiting the gut of *Ptiloglossa* larvae; (ii) there is no additional ecological filtering of the provisions once ingested. Larvae of several other solitary bees, as well as honey bee larvae, appear to be similar in these respects (Martinson et al., 2012; McFrederick et al., 2014, 2017; Kapheim et al., 2021). A strategy of suppressing bacterial colonization makes sense in light of the fact that most developing solitary bee larvae do not defecate until after they have completed feeding (Danforth et al., 2019); it may be too risky to allow microbial proliferation without a way of expelling excess cells. In contrast, we do observe a strong difference between provisions and whole-body larval microbiomes in *Crawfordapis*, mediated by the presence of *Wolbachia* in larvae. *Wolbachia* is also abundant in adult *Crawfordapis*. *Wolbachia* is fairly common in temperate bees (Gerth et al., 2015), and in at least some tropical bees such as *Megalopta centralis* (McFrederick et al., 2014).

Although dominated by lactobacilli, diphaglossine bee brood cells do harbor a variety of rarer bacteria. *Saccharibacter* is sporadically abundant in *Ptiloglossa* brood cells and adults, and in *Agave* nectar. *Saccharibacter* and other aerobic, acid-tolerant, and osmophilic Acetobacteraceae are commonly found in insects with sugar-rich diets (Crotti et al., 2010), on pollen (Jojima et al., 2004), and in the crop (Handy et al., 2023) and pollen provisions of bees (McFrederick et al., 2012). Fungal sequences are also present, but at very low relative abundances, in shotgun metagenomes from *Ptiloglossa* larval provisions. The lack of a substantial fungal community contrasts with earlier reports of yeasts in neotropical *Ptiloglossa* brood cells (Roberts, 1971; Batra et al., 1973). One possible explanation is that the presence of yeasts differs between *Ptiloglossa* in temperate deserts (studied here) versus tropical forests. Another explanation, which cannot be fully excluded, is a technical bias against fungi. However, our mock community metagenome harbors more yeast sequences than expected, and the DNA extraction protocol we used is similar to that used for fungal sequencing in sourdough starters, soil and other habitats (Rousk et al., 2010; Landis et al., 2021).

Although similar to related diphaglossines and to other solitary bee groups in terms of composition and host specificity (Figure 2; McFrederick et al., 2013, 2017; Kapheim et al., 2021), the abundance and activity of *Ptiloglossa* brood cell microbiomes appear to be unusual. First, larval provisions of *Ptiloglossa* have a much higher ratio of bacterial to plant DNA as compared with several other ground-nesting solitary bee species. Second, they are consistently observed to exhibit strong fermentation odors, which are only rarely reported from healthy brood cells of solitary bees. These two lines of evidence suggest that there has been a quantitative, but not qualitative, microbiome shift within diphaglossine bees. As more abundant beneficial microbes will generally confer stronger benefits to hosts (up to a point; Hammer et al., 2019), this shift may constitute a functionally novel form of symbiosis in *Ptiloglossa*.

Novel host-microbe symbioses often evolve through a process analogous to domestication, with capture and vertical transmission of host-restricted symbionts (Moran et al., 2019; Ganesan et al., 2022). Humans also domesticate microbes, propagating starter cultures used for fermentation of certain foods and beverages (Gibbons and Rinker, 2015; Steensels et al., 2019). In contrast, *Ptiloglossa* appear to culture undomesticated bacteria in their larval provisions. If confirmed, this result would demonstrate that quantitative microbiome shifts can evolve without changes in host specialization. A caveat is that symbiont domestication can occur rapidly (Stallforth et al., 2013; Bodinaku et al., 2019), without concomitant changes in 16S rRNA gene sequences. Another caveat is that our results are limited to *Ptiloglossa arizonensis*. The common ancestor of *Ptiloglossa* was most likely a tropical species, given that most extant diversity is in the tropics and subtropics (Michener, 2007), and the sister genus *Crawfordapis* (following, Velez-Ruiz, 2015) is also tropical. Hence it is not yet clear whether the undomesticated nature of *P. arizonensis* brood cell symbionts is ancestral or derived (e.g., related to adaptation to desert environments).

Our results suggest that the unusually fermentative brood cell microbiomes of *Ptiloglossa* evolved not through domestication, but simply by modification of the culturing environment. This path to symbiotic novelty has parallels with animals such as *Riptortus* bugs, which use modified gut structures to enrich non-host-specialized *Burkholderia* from the diet (Kikuchi et al., 2007; Ohbayashi et al., 2015). Some other animals behaviorally modify their environment to promote the growth of undomesticated “crops” (Zhu et al., 2016; Selden and Putz, 2022). Analogously, spontaneous (or natural) fermentation of certain foods and beverages relies on wild, undomesticated microbes. Lactobacilli often participate in this process; for example, *Apilactobacillus micheneri* (strain 11D in Figure 7) is a dominant member of the bacterial community in kôso, a fermented vegetable drink (Chiou et al., 2018). What traits enable *Ptiloglossa* to culture *Apilactobacillus* at high densities in their brood cells? Facilitated by waterproof brood cell linings, colletid bees in general, and *Ptiloglossa* in particular, tend to have more liquid larval provisions than other solitary bees (Roberts, 1971; Rozen, 1984; Almeida, 2008; Cane and Love, 2021). We hypothesize that this relatively high water content may facilitate microbial growth. How exactly highly liquid provisions are achieved is not known, but one possibility is that *Ptiloglossa* collect a large volume of nectar relative to pollen. *Ptiloglossa* often forage from flowers (like *Agave*) that produce large quantities of nectar (see Natural History). Another possibility is that by foraging in the early morning, *Ptiloglossa* collect particularly dilute nectar. Nectar is generally more dilute when first produced (Cane and Love, 2021).

As hypothesized by Roberts (1971), high bacterial densities in larval food likely benefit *Ptiloglossa* development. The elevated ratio of bacterial to plant DNA suggests that bacterial biomass is a major nutrient source for larvae, alongside pollen and nectar. Although almost all bee species are herbivorous (Michener, 2007), *Ptiloglossa* may have commonalities with insects that derive nutrition from microbes, such as *Drosophila* and dung beetles (Markow and O’Grady, 2008; Holter, 2016). Pollen and nectar contain all of the nutrients required for bee development (Roulston and Cane, 2000), but a “brewing” strategy could be economical for *Ptiloglossa*, a bee that almost exclusively forages from just before, to just after dawn. If bacteria upgrade the nutritional quality of the provisions, *Ptiloglossa* females may be able to rear more offspring despite a highly constrained foraging window. Brood cell bacteria could also play a role in defense. Ground-nesting, immature Hymenoptera (and their food) are vulnerable to attack by soil-borne microbes. Hence, both endogenous and bacterially based defenses are common (Kaltenpoth et al., 2005; Fernández-Marín et al., 2009; Strohm et al., 2019). For *Ptiloglossa*, the organic acids (and potentially ethanol) resulting from *Apilactobacillus* fermentation may suppress microbial invaders (McFrederick et al., 2018; Vuong and McFrederick, 2019) analogously to the anti-spoilage properties of lacto-fermented food. Indeed, despite the common presence of fungi in nectar and soil, fungal growth in *Ptiloglossa* brood cells appears to be kept to a minimum. Enrichment for lactobacilli occurs even before eggs are laid, potentially acting as a prophylactic antimicrobial defense for offspring, as occurs in some animals (Flórez et al., 2017; Kerwin et al., 2019).

To understand whether the diphaglossine bee brood cell symbiosis constitutes a mutualism, effects on bacterial fitness also need to be determined. Many insects have mechanisms to transmit symbionts vertically (Buchner, 1965), which more closely align fitness interests between hosts and symbionts (Ewald, 1987; Sachs et al., 2004). Many food fermentation practices also involve the reuse of starters or culture vessels, allowing domestication to occur. But, while the diphaglossine bee brood cell is clearly a highly favorable environment for local and short-term growth of lactobacilli, the bacteria may ultimately be digested by the larva or otherwise fail to escape alive. Indeed, lactobacilli consumed by larvae do not persist internally through the prepupal and pupal stages; we hypothesize that adults acquire them anew each generation from flowers. Other mechanisms by which bees could propagate lactobacilli need to be tested but seem unlikely. In theory, adults emerging from brood cells could acquire lactobacilli externally. But first, lactobacilli would need to survive for potentially several months *ex vivo*, as *Ptiloglossa arizonensis*, like many bees, overwinter as post-defecating prepupae (Rozen, 1984; Michener, 2007). Opportunities for emerging adults to contact residual lactobacilli in the brood cell are also limited. Postdefecating larvae of most diphaglossines pierce the cell lining such that feces drains into the soil; once the cocoon is spun, there is little direct exposure to remaining fecal material or the cell lining (Rozen, 1984).

If the dead-end hypothesis is correct, it implies a strong contrast with horizontally transmitted mutualisms in which symbionts benefit from their associations with hosts. For example, in the legume-rhizobia and bobtail squid-*Vibrio* mutualisms, hosts release the symbionts they culture back into the environment in large numbers (Lee and Ruby, 1994; Simms and Taylor, 2002). Indeed, adult diphaglossine bees harbor lactobacilli in their gut, and are likely important for their dispersal and persistence in the bee-flower niche. But the brood cell association may be more exploitative, with little to no long-term benefit to the lactobacilli.

In sum, the nesting biology of these bees appears to create favorable conditions for spontaneous fermentation or “brewing” of generalist lactobacilli: underground, temperature-stable brood cells; maintenance of high water content in provisions; suitable sugars (especially fructose); protection from contamination by the cellophane-like cell lining; open cells, possibly to allow venting; and, potentially, pre-enrichment of lactobacilli in the adult crop. Given the consistency of brood cell fermentation, particularly in *Ptiloglossa*, the bees likely benefit from culturing lactobacilli. On the other hand, lactobacilli may not benefit from being cultured, as they seem unable to escape the brood cell. While many details remain speculative, our findings provide an initial picture of the microbiology and ecology of a remarkable feat of fermentation.

## Data availability statement

The data presented in the study are deposited in the NCBI SRA repository, accession number PRJNA925568.

## Author contributions

TH, JK, QM, SB, and BD designed the study. JK, QM, WW, and BD helped acquire grant funding. TH, JK, SB, and BD conducted fieldwork. TH, JK, MA-G, and LG conducted labwork. TH and MA-G performed bioinformatic and statistical analyses. TH created the figures and drafted the manuscript, which was then read and edited by all authors. All authors contributed to discussion and interpretation of results.

## Funding

This work was supported by NSF DEB 1929572 Simons Foundation 429440 USDA-AFRI grant 1018839.

## Conflict of interest

The authors declare that the research was conducted in the absence of any commercial or financial relationships that could be construed as a potential conflict of interest.

## Publisher’s note

All claims expressed in this article are solely those of the authors and do not necessarily represent those of their affiliated organizations, or those of the publisher, the editors and the reviewers. Any product that may be evaluated in this article, or claim that may be made by its manufacturer, is not guaranteed or endorsed by the publisher.

## Footnotes

^1^http://iqtree.cibiv.univie.ac.at/

^2^https://doi.org/10.1093/nar/gkab301
