# Supplementary material for: Bee breweries: The unusually fermentative, lactobacilli-dominated brood cell microbiomes of cellophane bees
Source: Front Microbiol. 2023 Apr 5;14:1114849. doi: 10.3389/fmicb.2023.1114849 (PMC10113673; doi:10.3389/fmicb.2023.1114849)
Supplement: Supplementary file 1 [file Data_Sheet_1.docx]

**Supplemental Methods for Hammer et al. “Bee breweries: the unusually fermentative, lactobacilli-dominated brood cell microbiomes of cellophane bees”**

*Additional detail for 16S rRNA amplicon library prep*

Prior to PCR, we added partial Illumina sequencing adapters and unique 8-mer barcodes into each gDNA sample along with the 16S rRNA primers. We then added Phusion MasterMix (Thermo Scientific) and performed 25 cycles of PCR in 25 µl reaction per sample with a 52 °C annealing temperature. We cleaned PCR products with exonuclease and shrimp alkaline phosphatase. We then performed a second PCR using 1 µl of cleaned PCR product per sample as the template and primers to complete the Illumina sequencing construct: PCR2F (5’-CAAGCAGAAGACGGCATACGAGATCGGTCTCGGCATTCCTGC-3’) and PCR2R (5’-AATGATACGGCGACCACCGAGATCTACACTCTTTCCCTACACGACG-3’). We used the same cycling conditions for both PCRs: An initial 94 °C denaturing step for 3 min followed by 25 cycles of 94 °C for 45 s, 52 °C for 1 min, and 72 °C for 90 s.

*Library prep for shotgun metagenomics*

To construct metagenomic libraries, we prepared a reaction mix in a PCR plate containing DNA, FX Buffer, and nuclease-free water. To conduct enzymatic fragmentation, we incubated at 32 °C for 20 minutes followed by at 30 minute incubation at 65 °C to denature remaining enzymes. We then prepared the ligation master mix using the ligation buffer, DNA ligase, and nuclease-free water. We added 45 µl of ligation master mix to each sample and proceeded to cleanup using AMPure XP beads. Next, we amplified the libraries with 6 PCR cycles in 50 ul reaction per sample, using the supplied master mix and primer mix. Thermocycler conditions were as follows: 4 °C for 1 min, enzymatic fragmentation at 32 °C for 12 mins, 65 °C for 30 mins, and a 4 °C hold. We then eluted 23.5 µl of each library into 26 µl of buffer EB and stored the libraries in a DNA LoBind plate.

*Additional detail for amplicon data analysis*

To decide how many bases to truncate and trim, we selected sequences with a Phred quality score between 20 and 37 at the bottom of the box. For *Caupolicana* libraries, we truncated forward reads at 141 bp and 128 bp for reverse reads; trimming was not necessary. For *Ptiloglossa* libraries, we truncated forward reads at 203 bp, 163 bp for reverse reads, and we left-trimmed reverse reads at 18 bp. For *Crawfordapis* libraries, we truncated forward reads at 210 bp, 228 bp for reverse reads, and we left- and right-trimmed reverse reads at 20 bp.

*References for the Supplemental Methods*

Caporaso JG, Lauber CL, Walters WA, Berg-Lyons D, Huntley J, Fierer N, Owens SM, Betley J, Fraser L, Bauer M, & Gormley N. (2012) Ultra-high-throughput microbial community analysis on the Illumina HiSeq and MiSeq platforms. *The ISME Journal* 6: 1621-4.

Quince C, Lanzen A, Davenport RJ, & Turnbaugh PJ. (2011) Removing noise from pyrosequenced amplicons. *BMC Bioinformatics* 12: 38.
