# Supplementary material for: Bee breweries: The unusually fermentative, lactobacilli-dominated brood cell microbiomes of cellophane bees
Source: Front Microbiol. 2023 Apr 5;14:1114849. doi: 10.3389/fmicb.2023.1114849 (PMC10113673; doi:10.3389/fmicb.2023.1114849)
Supplement: Supplementary file 2 [file Table_1.docx]

**Supplemental Table for Hammer et al. “Bee breweries: the unusually fermentative, lactobacilli-dominated brood cell microbiomes of cellophane bees”**

| **Dataset** | **Primer pair** | **Target region(s)** |
| --- | --- | --- |
| *Caupolicana* | 515F (5’-GTGYCAGCMGCCGCGGTAA-3’), 806R (5’-GGACTACNVGGGTWTCTAAT-3’) | V4 |
| *Ptiloglossa* | 799F (5’-CMGGATTAGATACCCKGG-3’), 1115R (5’-AGGGTTGCGCTCGTTG-3’) | V5-V6 |
| *Crawfordapis* | 515F (5’-GTGCCAGCMGCCGCGGTAA-3’), 926R (5’-CCGYCAATTYMTTTRAGTTT-3’) | V4-V5 |

Table S1. Primers used for the three 16S rRNA amplicon datasets (Quince et al. 2011, Caporaso et al. 2012).
